# West syndrome followed by juvenile myoclonic epilepsy: a coincidental occurrence?

**DOI:** 10.1186/1471-2377-13-48

**Published:** 2013-05-24

**Authors:** Salvatore Mangano, Rosaria Nardello, Gabriele Tripi, Giuliana Giordano, Chiara Spitaleri, Giuseppa Renata Mangano, Antonina Fontana

**Affiliations:** 1 Department of Sciences for Health Promotion and Mother and Child Care "G. D'Alessandro", Child Neuropsychiatry Unit, Università di Palermo, Palermo, Italy; 2 Psychology Department, Università di Palermo, Palermo, Italy

**Keywords:** West syndrome, Juvenile myoclonic epilepsy, Epilepsy evolution, Genetic predisposition, Hairy elbows syndrome

## Abstract

**Background:**

West syndrome is an age-dependent epilepsy with onset peak in the first year of life whose aetiology may be symptomatic or cryptogenic. Long-term cognitive and neurological prognosis is usually poor and seizure outcome is also variable. Over the past two decades a few patients with favourable cognitive outcome and with total recovery from seizures were identified among the cryptogenic group suggesting an idiopathic aetiology. Recent research has described two children with idiopathic WS who later developed a childhood absence epilepsy.

**Case presentation:**

We reviewed the medical records of patients with West syndrome admitted to the our Child Neuropsychiatry Unit in the last 15 years in order to know the clinical evolution of infantile spasms.

We report a child with West syndrome with onset at 8 months of age followed by some clusters of bilateral, arrhythmic myoclonic jerks of the upper limbs, mainly on awakening, synchronous with the generalized discharges of 4 Hz spike-wave occurring at 12 years of age and by co-occurrence of a later generalized tonic-clonic seizure at 14 years and four months, both sensitive to Levetiracetam suggesting a juvenile myoclonic epilepsy.

**Conclusions:**

This unusual evolution, never previously reported, suggests that both electroclinical features mentioned above may share some pathophysiological processes genetically determined which produce a susceptibility to seizure and emphasizes that the transition between different age-related epileptic phenotypes may involve also the West syndrome.

## Background

West syndrome (WS) is an age-dependent epilepsy with onset peak in the first year of life, characterized by epileptic spasms occurring in clusters, psychomotor delay or deterioration, and a specific interictal electroencephalogram (EEG) pattern known as hypsarrhythmia [1].

According to the ILAE [1] classification of epilepsies and epileptic syndromes, the aetiology of WS may be symptomatic (80%) or cryptogenic (20%). An idiopathic aetiology was also proposed when there is a normal development before onset of symmetric spasms, without any other kind of seizure, associated with normal neuroimaging, the lack of focal interictal or ictal EEG abnormalities, no identified etiologic factors except for a general family predisposition to epilepsy, and a good neurodevelopmental outcome [2-4].

Usually, WS is associated with a poor long-term cognitive and neurological prognosis and only 9%-24% of patients had normal intelligence or slightly impaired intelligence [5,6].

Seizure outcome is also variable. A detailed long-term study reported that among 143/147 surviving children the spasms stopped before the age of 3 years. Forty-eight patients were seizure-free, 11 had infrequent seizures (1–2 a year), 19 patients had 3–10 seizures a year, 13 patients had 1–2 seizures a month, 16 patients had 3–6 seizures a month, and 26 had seizures daily or almost daily [6]. The seizures were partial or focal and often secondarily generalized in 27% of the patients, and Lennox-Gastaut syndrome was found in 18% of the patients [6].

Recent research described two children who at the age of 8 and 3 months developed “idiopathic” WS followed, at the age of 6 and 4 years respectively, by childhood absence epilepsy (CAE) [7].

We report a child with WS with onset at 6–8 months of age followed by myoclonic seizures at 12 years of age, and later, at 14 years and four months, by a generalized tonic-clonic seizures. This unusual evolution, never previously reported, suggested that both electroclinical features may share some pathophysiological processes genetically determined which produce a susceptibility to seizure.

## Case presentation

This study received the informed consent of the patient's parents.

The proband, a male adolescent, was the second offspring born to healthy unrelated parents after an uneventful pregnancy and spontaneous delivery. His birth weight was 3820 g, height 54 cm, and head circumference 35,5 cm. The family history is remarkable for the occurrence in three generations in the maternal line of two fourth-degree relatives with early onset epilepsy remitted during childhood, two adult second -degree relatives with juvenile onset epilepsy still receiving AEDs, and five fourth-degree relatives aged 3–16 years with epilepsy not further defined. All adult members are engaged in a work activity.

Personal history before onset of spasms revealed a mild psychomotor delay. The infant was referred to our Department at the age of 8 months because he showed clusters of symmetric spasms occurring 3-4/die, mainly on awakening, lasting 2 months with an increased frequency in the last week, never associated with other seizure types.

On admission, neurological and psychomotor examination revealed mild hypotonia of lower limbs, difficulties of unsupported sitting, and impairment in social interactions.

**Figure 1:**
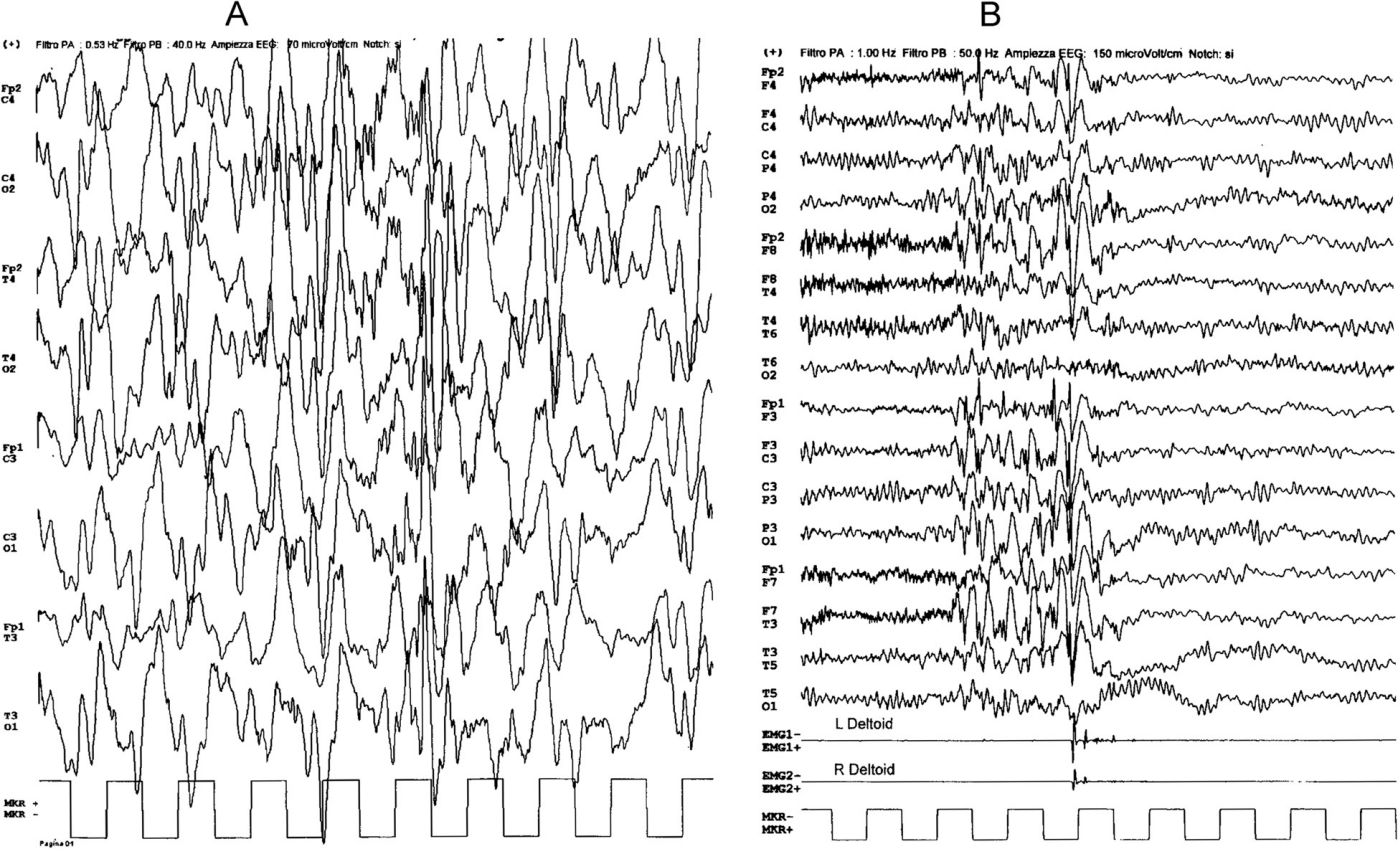
A. Interictal EEG displays diffuse and asynchronous high voltage spikes and slow waves suggesting the hypsarrhythmic pattern. B. Ictal EEG shows a myoclonic jerk of the upper limbs, synchronous with the generalized discharges of 4 Hz spike-wave.

Interictal EEG recording during n-REM sleep and wakefulness displayed diffuse and asynchronous high voltage spikes and slow waves suggesting the hypsarrhythmic pattern (Figure 1A).

The clinical and EEG data suggested West syndrome diagnosis which required an AED treatment. Since Valproate and Nitrazepam were ineffective, 14 days later ACTH was started in add-on inducing the enduring seizure control, hypsarrhythmia resolution within 10 days without further relapse, and improvement in emotional and social interaction.

Then, both motor and mental abilities developed but below the normal range.

At 1 year of age increasingly long and thick hairs on both the elbow regions were observed. The elbow skin biopsy showed hypertrophy of the hair follicle without epidermal and dermal layer abnormalities.

**Figure 2:**
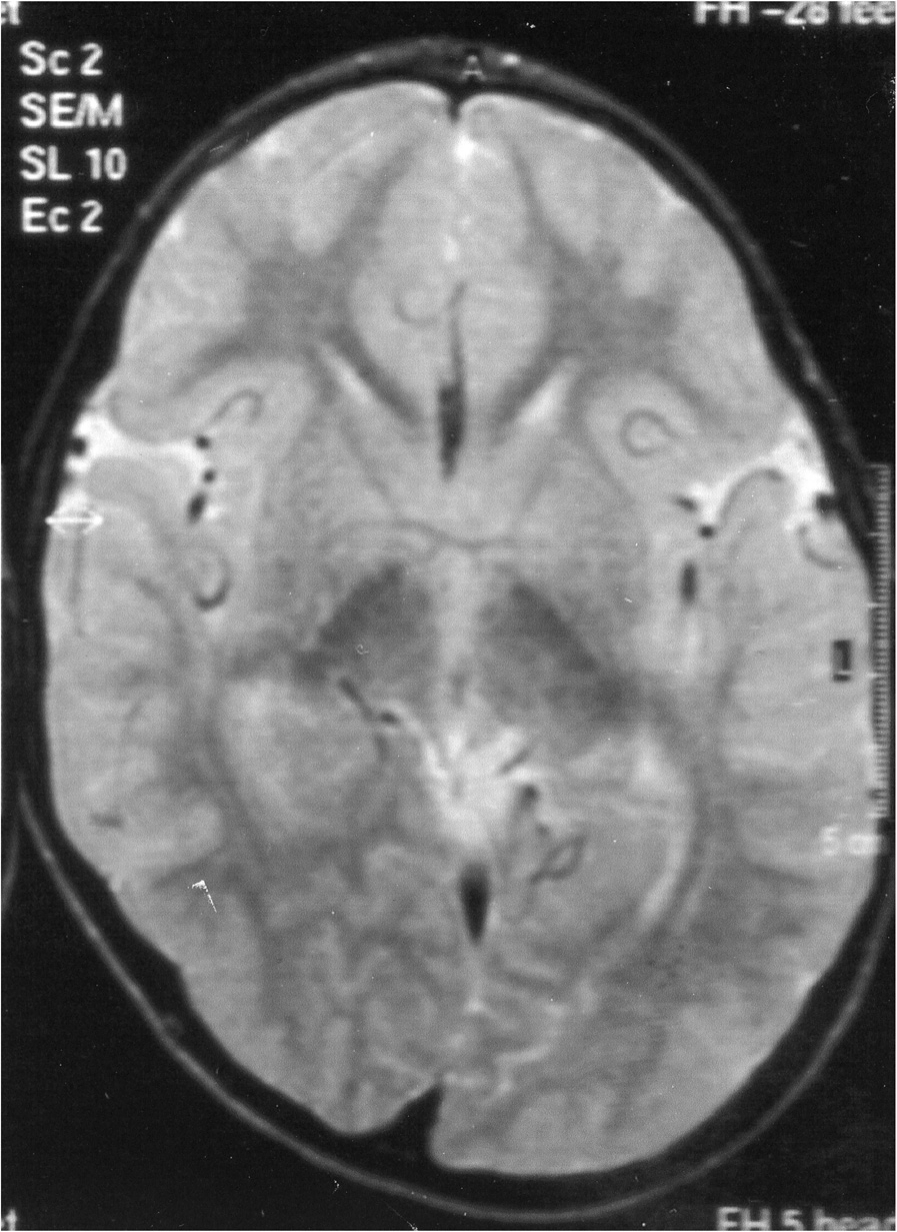
Axial PD-weighted magnetic resonance imaging of the Brain, performed at 3 years of age, shows hemispheric asymmetry with size increase of left posterior hemisphere.

Brain magnetic resonance imaging (MRI), performed at 3 years, showed a size increase of left posterior hemisphere (Figure 2).

Chromosome analysis, performed upon cells obtained from peripheral lymphocytes and skin fibroblasts revealed a XY karyotype without mosaicism.

The sequencing of the aristaless related homeobox *(ARX)* gene, molecular analysis of sub-telomeric regions, and array comparative genomic hybridization did not show abnormal findings. Other laboratory and metabolic tests were normal.

At 4 years and 3 months he was seizure-free, untreated, quiet, smiling, and easily engaged in activities, but he played with simple toys, and exhibited a slow lexical development with phonetic-phonological difficulties. The intellectual assessment using Stanford - Binet Intelligence scale (Terman – Merrill, Form LM, 1960; OS Giunti, Firenze 1968) revealed an IQ 47.

During follow-up, the child did not show gross neurological deficits and the EEGs during n-REM sleep and wakefulness showed age appropriated background activity without abnormalities.

At 12 years of age he experienced some clusters of bilateral, arrhythmic myoclonic jerks of the upper limbs, mainly on awakening, synchronous with the generalized discharges of 4 Hz spike-wave (Figure 1B). The interictal EEG during wakefulness showed age appropriated background activity with infrequent generalized discharges of 4 Hz spike-wave during hyperventilation. Rarely, intermittent 13 Hz photic stimulation triggered myoclonic jerks associated with generalized discharges of 4 Hz spike-wave.

The increase of the seizure frequency, never associated with other seizure types, required Levetiracetam (LEV) treatment that induced a seizure control except when he was worried and/or sleep deprived.

At 14 years and four months on awakening he had a longer than usual cluster of myoclonic jerks with increasing amplitude and frequency which evolved into a generalized tonic-clonic seizure (GTCS).

His academic skills were always impaired and he received permanent educational support at school. At 13 years and 6 months intellectual assessment using the Wechsler Intelligence Scale for Children (WISC-III) showed a moderate-mild mental retardation [Full scale IQ 48 (C.I. 99% 43–59), Verbal IQ 60 (C.I. 99% 53–73), Performance IQ 48 (C.I. 99% 43–63)].

## Discussion

The clinical history of our patient has been characterized by the occurrence of the typical triad of West syndrome. The somatic abnormalities found in our patient suggested an additional diagnosis of “Hairy Elbows Syndrome”, a disorder with an unknown aetiology but frequently associated with intellectual disability and speech delay [8,9].

At the time of diagnosis, the size increase of the left posterior hemisphere and the early psychomotor delay suggested a symptomatic aetiology of WS. Nevertheless, the quick and complete remission of spasms, never associated with other seizure types, the disappearance of hypsarrhythmic pattern, followed by an improvement of psychomotor development after ACTH treatment, and the seizure-free interval without EEG abnormalities during sleep and wakefulness until 12 years of age, resulted in a more favourable prognosis than that previously hypothesized.

In addition, the parents observed since the neonatal period a mild developmental delay but they did never detected a significant and/or enduring psychomotor regression beyond the remission of the spasms suggesting an “epileptic encephalopathy” [10].

Therefore, it is likely that the current intellectual disabilities represent mainly the developmental step of the early psychomotor delay, which in association with the structural brain abnormalities result from a congenital disorder.

The coexistence of the “Hairy Elbows Syndrome” and the West syndrome has never been reported. Thus, the relationship between the two syndromes need further investigations since even the molecular analyses, including ARX gene, mainly involved in brain development, were negative in our patient. However, the favourable clinical course, the lack of other seizure types, and the family history of recurrence of different age-dependent epileptic syndromes further weaken the link between WS and HES [2-4,11] although a direct relationship between the two disorders can not be fully excluded.

The appearance at 12 years of age of myoclonic jerks of upper limbs synchronous with the typical generalized discharges of 4 Hz spike-wave, associated with uncomplicated clinical course, and the lack of other seizure types except the later GTCS, both sensitive to Levetiracetam, recalled an age-dependent epileptic syndrome.

We are prone to discern in the electroclinical feature of our patient, with the specific chronological sequence of the seizures above reported, the occurrence of the diagnostic criteria of JME recognized by the Classification of Epilepsies and Epileptic Syndromes [1] despite the abnormal brain MRI findings as brain lesions have previously been reported in patients with JME [12].

On the other hand the typical electroclinical phenotypes following the WS include focal and often secondarily generalized seizures and Lennox-Gastaut syndrome, whereas the myoclonic seizures associated with GTCS with late onset have not been described in previous follow-up studies [6].

However, from our point of view there is no obvious relationship between such presumed symptomatic spasms and JME, but it is striking to observe coexistence of two age-dependent epileptic syndromes in the same patient.

Recently, the transition between the more common age- dependent epilepsies has been documented and some overlapping pathophysiological processes and common genetic factors have been hypothesized [13].

Furthermore, the transition from WS to CAE has recently been described in two children [7]. Likewise, the clinical course of our patient might be read as a transition from an age-dependent epilepsy (WS) to another one (JME).

The linking between WS and the JME proves to be a complex relationship since to our knowledge common genetic alterations have not been reported in the literature.

As the variable etiology of infantile spasms, more than 200, lead to the same electroclinical feature it has been speculated that different clinical conditions may converge on a final common pathway [14]. In agreement with this view, it has been recently hypothesized that some types of epilepsy, including WS and JME, may depend on the dysfunction and on a specific susceptibility of a given neural system to epileptogenic factors (system epilepsy) [15].

It is likely that some genes other than those currently known or non-conventional genetic influences such as epigenetic, or environmental factors play a role in seizure predisposition.

Therefore, the co-occurrence of WS and JME in our patient may depend on the dysfunction of specific brain systems which to some extent overlap or influence each other. Thus, the usual occurrence of the seizures during periods of sleep-wake transitions in both syndromes may be a clinical trait of a common dysfunction present in WS and JME. Of course, future studies of functional neuroimaging could help to understand these relationships.

## Conclusion

In conclusion, our study raises the question regarding the incidence of the coexistence of these two epileptic syndromes, and indicates the need to further investigate the relationship between the two syndromes and to elucidate the pathogenetic mechanisms of the evolution of WS to JME as well as any common neurobiological and genetic substrate.

## Consent

Written informed consent was obtained from the patient for publication of this Case report and any accompanying images. A copy of the written consent is available for review by the Series Editor of this journal.

## Competing interests

The authors declare no conflict of interest.

## Authors’ contributions

SM participated in conceptualization of the study and in revising the manuscript. AF participated in conceptualization of the study and in interpretation of the neuropsychological data. CS participated in acquisition of the clinical data. GRM participated in acquisition of the neuropsychological data. RN participated in drafting the manuscript. GT participated in analysis and interpretation of EEG data. GG participated in acquisition of EEG data. All authors read and approved the final manuscript.

## Author information

SM: MD, Professor of Child Neuropsychiatry; RN: MD, PhD; GT: MD, PhD; GG: MD; CS: MD; GRM: PhD; AF: PsyD.

## Pre-publication history

The pre-publication history for this paper can be accessed here:

http://www.biomedcentral.com/1471-2377/13/48/prepub

## References

[B1] International League Against Epilepsy Proposal for revised classification of epilepsies and epileptic syndromes Epilepsia 1989 30 389 399 2502382 10.1111/j.1528-1157.1989.tb05316.x

[B2] Commission on Pediatric Epilepsy of the International League Against Epilepsy Workshop on infantile spasms Epilepsia 1992 33 195 9579929

[B3] Dulac O Plouin P Jambaque I Predicting favorable outcome in idiopathic west syndrome Epilepsia 1993 34 747 756 10.1111/j.1528-1157.1993.tb00457.x 8330588

[B4] Vigevano F Fusco L Cusmai R Claps D Ricci S Milani L The idiopathic form of West syndrome Epilepsia 1993 34 743 746 10.1111/j.1528-1157.1993.tb00456.x 8330587

[B5] Hrachovy RA Glaze DG Frost JD Jr A retrospective study of spontaneous remission and long-term outcome in patients with infantile spasms Epilepsia 1991 32 212 214 10.1111/j.1528-1157.1991.tb05246.x 1848513

[B6] Riikonen R Long-term outcome of west syndrome: a study of adults with a history of infantile spasms Epilepsia 1996 37 361 372 10.1111/j.1528-1157.1996.tb00573.x 8603642

[B7] Specchio N Trivisano M Vigevano F Fusco L Idiopathic west syndrome followed by childhood absence epilepsy Seizure 2010 19 597 601 10.1016/j.seizure.2010.07.016 20729098

[B8] Polizzi A Pavone P Ciancio E La Rosa C Sorge G Ruggieri M Hypertrichosis cubiti (hairy elbow syndrome): a clue to a malformation syndrome J Pediatr Endocrinol Metab 2005 18 1019 1025 16355816 10.1515/jpem.2005.18.10.1019

[B9] Nardello R Mangano S Fontana A Tripi G Didato MA Di Pace M Corsello G The hairy elbows syndrome: clinical and neuroradiological findings Pediatr Med Chir 2008 30 262 264 19320141

[B10] Berg AT Berkovic SF Brodie MJ Buchhalter J Cross JH van Emde Boas W Engel J French J Glauser TA Mathern GW Revised terminology and concepts for organization of seizures and epilepsies: report of the ILAE Commission on Classification and Terminology, 2005–2009 Epilepsia 2010 51 676 685 10.1111/j.1528-1167.2010.02522.x 20196795

[B11] Lux AL Osborne JP The influence of etiology upon ictal semiology, treatment decisions and long-term outcomes in infantile spasms and West syndrome Epilepsy Res 2006 70 Suppl 1 S77 S86 16828260 10.1016/j.eplepsyres.2006.01.017

[B12] Thomas P Genton P Gélisse P Medina M Serafini A Bureau M, Genton P, Dravet C, Delgado-Escueta A, Tassinari CA, Thomas P, Wolf P Juvenile myoclonic epilepsy Epileptic syndromes in infancy, childhood and adolescence 2012 5 Paris: John Libbey Eurotext Ltd 305 308

[B13] Koutroumanidis M Panayiotopoulos syndrome: an important electro-clinical example of benign childhood system epilepsy Epilepsia 2007 48 1044 1053 10.1111/j.1528-1167.2007.01096.x 17441996

[B14] Frost JD Hrachovy RA Pathogenesis of infantile spasms: a model based on developmental desynchronization J Clin Neurophysiol 2005 22 25 36 10.1097/01.WNP.0000149893.12678.44 15689710

[B15] Avanzini G Manganotti P Meletti S Moshé SL Panzica F Wolf P Capovilla G The system epilepsies: a pathophysiological hypothesis Epilepsia 2012 53 771 778 10.1111/j.1528-1167.2012.03462.x 22533642

